# Uncarboxylated osteocalcin promotes proliferation and metastasis of MDA-MB-231 cells through TGF-β/SMAD3 signaling pathway

**DOI:** 10.1186/s12860-022-00416-7

**Published:** 2022-04-12

**Authors:** Jiaojiao Xu, Luyao Ma, Danqing Wang, Jianhong Yang

**Affiliations:** 1 Medical School, University of Chinese Academy of Sciences, 19A Yuquan Road, Beijing, 100049 People’s Republic of China; 2 Institute of Molecular Rhythm and Metabolism, Guangzhou University of Chinese Medicine, Guangzhou, 510006 People’s Republic of China

**Keywords:** Breast cancer, Osteocalcin, TGF-β, Proliferation, Metastasis

## Abstract

**Background:**

Triple-negative breast cancer (TNBC) is the most severe type of breast cancer owing to its high heterogeneity, aggressiveness and lack of treatment. Studies have reported that uncarboxylated osteocalcin (GluOC) promotes the development of prostate and other cancers. Studies have also found elevated levels of serum osteocalcin in breast cancer patients with bone metastasis, and serum osteocalcin can be a marker of bone metastasis. However, whether GluOC promotes the development of TNBC and the related mechanisms need to be further clarified.

**Results:**

Our results revealed that GluOC is associated with the proliferation and metastasis of MDA-MB-231 cells. GluOC increased the viability and proliferation of MDA-MB-231 cells. In addition, GluOC enhanced the metastatic ability of MDA-MB-231 cells by promoting the expression of matrix metalloproteinase-2 (MMP2), matrix metalloproteinase-13 (MMP13), and vascular endothelial growth factor (VEGF) and inducing epithelial-mesenchymal transition (EMT). We also found that GluOC upregulated the expression of interleukin-8 (IL-8) and parathyroid hormone-related protein (PTHrP) genes in MDA-MB-231 breast cancer cells. Moreover, the promoting effect of GluOC was reversed in MDA-MB-231 breast cancer cells treated with specific inhibitor of SMAD3 (SIS3), a SMAD3 phosphorylation inhibitor.

**Conclusion:**

Our research proved for the first time that GluOC facilitates the proliferation and metastasis of MDA-MB-231 cells by accelerating the transforming growth factor-β (TGF-β)/SMAD3 signaling pathway. Moreover, GluOC also promotes the gene expression of IL-8 and PTHrP. Both IL-8 and PTHrP can act as osteolytic factors in breast cancer cells. This study indicates that GluOC may be a useful target for preventing TNBC bone metastasis.

**Supplementary Information:**

The online version contains supplementary material available at 10.1186/s12860-022-00416-7.

## Background

Breast cancer (BC) has become most common cancer among women [1]. The World Health Organization's International Agency for Research on Cancer (IARC) 2018 GLOBOCAN report ranked breast cancer as the most common cancer among females (representing 24% of female cancer cases). The occurrence of breast cancer among Chinese women is approximately 41/100,000, and the incidence is increasing year by year. Among all breast cancers, the occurrence of triple-negative breast cancer (TNBC) is approximately 15% ~ 20% [2]. TNBC is estrogen receptor (ER), progesterone receptor (PR) and human epidermal growth factor receptor-2 (HER2) negative breast cancer, is not sensitive to common endocrine therapy and targeted therapy, has a poor prognosis, and is prone to early recurrence and metastasis [3]. Patients with metastatic TNBC have a survival period of approximately 18 months, which is lower than that of patients with other types of breast cancer, for whom the survival may be longer than 5 years [4]. To date, the treatment of TNBC is still unsatisfactory; therefore, there is an urgent need to understand the mechanisms of TNBC invasion and metastasis, which can help identify new targets and screen drugs.

Tumor metastasis is a complex, multistage process. In this process, matrix metalloproteinases (MMPs), which participate in pathways leading to the development of metastasis, degrade and remodel the extracellular matrix. Vascular endothelial growth factor (VEGF) promotes the development of vascular endothelial cells to form new tumor blood vessels to provide nutrition and nutrients for their growth and metastasis and thus accelerates the occurrence and development of breast cancer [5]. Epithelial-mesenchymal transition (EMT) endows cancer cells with a mesenchymal phenotype to achieve mobility as well as additional properties and promotes invasion, migration and subsequent spread [6]. Breast cancer cells, by expressing parathyroid hormone-related protein (PTHrP) and interleukin-8 (IL-8), promote the activity of osteoclasts, which are responsible for perturbing bone homeostasis [7]. Jie Wang et al. found that the migration, invasion and bone metastasis of MDA-MB-231 cells can be prevented by silencing the bone sialoprotein (BSP) gene [8]. Shuang Qu et al. clarified that Osterix stimulates MDA-MB-231 migration and angiogenesis by upregulating S100A4 [9]. However, the mechanism of bone metastasis in TNBC needs to be further studied.

The regulatory cytokine transforming growth factor beta (TGF-β) is a factor of the cytokine network and has the function of regulating biological growth [10]. The receptor complex is activated, and TGF-β signaling is ultimately initiated through the specific C-terminal phosphorylation of SMADs (R-SMADs), SMAD2 and SMAD3 [11]. TNBC cells also produce SMAD2/3 and SMAD4, which lead to metastasis and angiogenesis [12]. TGF-β can not only play a role in local invasion, but increasing evidence has also shown that TGF-β has an essential effect in cancer cell metastasis by promoting the cancer cell proliferation, EMT and other processes. Hu Z et al. found that the TGF-β signaling pathway can be exploited to develop a PC-3 (human prostatic cancer cell line) bearing mouse model of bone metastasis of prostate cancer [13]. Kakonen et al. also discovered that TGF-β may stimulate osteolytic cytokines (PTHrP) released by cancer cells and induce osteolytic bone metastasis [14]. Brewster AM et al. reported that TNBC signaling pathway therapies include those targeting PARP, PI3K/mTOR, JAK/STAT, Notch, etc. [15]. However, the signaling pathways related to bone metastasis in TNBC still need to be further explored.

Osteocalcin is expressed and secreted by osteoblasts [16]. After protein synthesis, its three glutamic acid residues can be carboxylated to varying degrees [17, 18]. Osteocalcin can take on the forms of completely carboxylated osteocalcin and incompletely carboxylated osteocalcin. Incompletely carboxylated osteocalcin includes uncarboxylated osteocalcin (GluOC) [19]. Increasing evidence has suggested that GluOC regulates glucose metabolism, neural development, and the male reproductive track, and has a relationship with tumorigenesis [20]. Ye P [21] and Kayed H et al. [22] found that GluOC has a tumor-promoting effect on the human prostate cancer cell line PC-3 and pancreatic cancer cells. Kyung-Hun Lee et al. clarified that the level of circulating osteocalcin-positive cells can be used as a predictor of early bone metastasis in patients with breast cancer metastasis [23]. Salem AM et al. also suggested that serum osteocalcin content in breast cancer patients and bone metastasis patients was increased remarkably compared with that in the control group [24]. However, the mechanism by which GluOC affects the proliferation and bone metastasis of TNBC remains unclear.

Here, we selected MDA-MB-231 cells, which are highly metastatic, and MCF7 cells, which are nonmetastatic, as research objects to explore the impact of GluOC on TNBC metastasis. The results demonstrated that GluOC can significantly promote the proliferation and migration of MDA-MB-231 cells via the TGF-β/SMAD3 signaling pathway, but there was no obvious influence on MCF7 breast cancer cells. These research results showed that GluOC has an indispensable role in the proliferation and migration of MDA-MB-231 cells. In addition, our study also emphasizes that GluOC is a novel target for the prevention and/or treatment of TNBC bone metastasis.

## Materials and methods

### Reagents and antibodies

Antibodies against proliferating cell nuclear antigen (PCNA, cat. no. ab92552, Abcam), matrix metallopeptidase 2 (MMP2, cat. no. ab2536, Abcam), Vimentin (cat. no. ab92547, Abcam), N-cadherin (cat. no. ab76011, Abcam), Snail (cat. no. ab216347, Abcam), E-cadherin (cat. no. ab231303, Abcam), TGF-β (cat. no. ab215715, Abcam), SMAD2 + SMAD3 (SMAD2/3, cat. no. ab202445, Abcam), p-SMAD3 (cat. no. ab52903, Abcam), cyclin D1 (cat. no. CPA4263, Cohesion), and β-actin (cat. no. 13E5, Cell Signaling Technology, Inc.) and goat anti-rabbit IgG(H + L)-HRP (cat. no. S0101, Lablead Biotech, Co. Ltd), goat anti-mouse IgG(H + L)-HRP (cat. no. S0100, Lablead Biotech, Co. Ltd), 1% crystal purple solution (cat. no. G1064, Solarbio), Native Lysis Buffer (cat. no. R0030, Solarbio), a BCA protein concentration assay kit (cat. no. B5000; Beijing Lablead Biotech, Co., Ltd.), and trypsin cell digestive fluid (0.25% trypsin, cat. no. C0202, Beyotime) were used in this study.

### Cell culture

MDA-MB-231 is a human TNBC cell line that is highly invasive and metastatic. MCF7 is a human ER-positive breast cancer cell line that is a nonmetastatic and has low invasiveness. Cells were purchased from the Type Culture Collection of the Chinese Academy of Sciences, Shanghai, China. The cells were cultured in Dulbecco’s modified Eagle’s medium (DMEM, Gibco) containing 10% fetal bovine serum (FBS, cat. no. 10270–106, Gibco). After that, the cells were cultured in a moist 37 °C incubator with 5% CO_2_. Thereafter, the medium was replenished every 24 h. Finally, the cells were cultured in serum-free medium for 12 h before treatment with GluOC.

### Cell viability assay

A Cell Counting Kit-8 (CCK8, cat. no. CK04, Dojindo Molecular Technologies, Inc.) was used to quantify cell viability. Cells were inoculated at 1 × 10^4^ cells/well into 96-well plates. After the number of cancer cells reached 60%, the cells were cultured in serum-free medium and GluOC at different doses (10, 20, 40, 100 and 160 ng/ml) for 4 h, 8 h, 12 h, 24 h, 48 h, 72 h, 96 h and 120 h. Room temperature phosphate buffer saline (PBS) was used to clean the cells, and CCK8 solution (10 μL) was added to 6-well plates, which were incubated in the incubator for 1 h. The absorbance was measured at 450 nm with an automated microplate reader (Synergy H1; Biotek Instruments, Inc.).

### Wound healing assay

The wound healing assay was applied to calculate the migration capacity of cancer cells. The cells were placed in a 6-well plate and incubated until they were approximately 90% confluent. A 10 µl micropipette tip was used to scrape the cell monolayer, and the debris was rinsed four times with PBS. The cells were placed in culture medium containing different concentrations of GluOC (10, 20, 40, 100 and 160 ng/ml), incubated for 24 h, and then observed with an inverted microscope (cat. no. DP71, OLYMPUS). Cell images were taken at 100 × magnification. Three random areas with cells were selected, and the migration rate was calculated. Cells in culture medium without GluOC were used as vector controls. Wound area can be calculated by manually tracing the cell-free area in images using ImageJ software version 6 (National Institutes of Health). Under normal conditions, the wound area will decrease over the time. The migration rate can be expressed as the percentage of area reduction of wound closure, which increases as cells migrate over time. Wound closure %: (A_t=0 h_ – A_t=24 h_) / A_t=oh_ × 100%. Where A_t = 0 h_ is the area of the wound measured immediately after scratching (time zero), and A_t=24 h_ is the area of the wound measured 24 h after the scratch is performed. All experimental data are from three independent experiments.

### Cell migration assay

Migration was tested as previously described using a transwell chamber (6.5 mm in diameter, 8 μM pore-size, Corning Costar, Cambridge, MA) [25]. The cells were serum-free for 12 h, rinsed four times with PBS, and then recultivated in a serum-free medium. The cell density in the upper compartment was 1 × 10^4^. Culture medium containing 10% FBS was added to the lower compartment. The 24-well plate was placed in the incubator for culture for 12 h. Different concentrations of GluOC were added for culture for 24 h, and the samples were washed 3 times with PBS, fixed with 10% formaldehyde solution for 30 min, dyed with 1% crystal violet solution and incubated for 30 min. After air drying, the cells above the chamber were wiped off with a cotton swab, and pictures were taken using an inverted microscope (cat. no. MK3, Multiskan). Cell images were taken at 100 × magnification (avoiding the edge of the chamber, to avoid distorting the results with edge effects).

### SIS3 treatment

Specific inhibitor of SMAD3 (SIS3) is a specific inhibitor of SMAD3 phosphorylation [26]. SIS3 powder (cat. no. T3636, TargetMoL) was dissolved in 100% dimethyl sulfoxide (DMSO, cat. no. D6258, Macklin Biochemical Co., Ltd.) and prepared into a solution with a final concentration of 1 mM. MDA-MB-231 cells were grown to approximately 90% confluency in a 6-well plate, and gently washed with PBS four times. Subsequently, the cultures were grouped into three different culture conditions: (A) control; (B) SIS3 (10 μM); and (C) SIS3 (10 μM) + GluOC (40, 100, 160 ng/mL). After continuous culture for 24 h, the effect of SIS3 on the proliferation and migration of breast cancer cells was detected by western blotting.

### Western blot analysis

The breast cancer cells were gently rinsed four times with 1 × PBS and harvested in cell lysis buffer containing 1 mM phenylmethanesulfonyl fluoride (PMSF, cat. no. P0100, Solarbio). The cells were lysed on ice for 10 min. The whole cell lysate was centrifuged at 1,2000 g for 10 min at 4 °C, and the supernatant was collected for subsequent applications. Equal amounts of protein were isolated by 12% SDS-PAGE, and the proteins were transferred to polyvinylidene fluoride (PVDF, cat. no. ISEQ00010, Merck Millipore, Co., Ltd.) membranes by membrane electrophoresis for 2 h. The membranes were then sealed with TBS‑5% Tween‑20 (TBST) containing 5% skim milk at room temperature for 2 h to prevent binding of nonspecific antibodies. The dilutions of various primary antibodies were applied, followed by overnight incubation at 4 °C at 1:1000 concentration as recommended by the supplier. Then, the PVDF membranes were washed three times with TBST (10 mM Tris, 10 mM NaCl, 0.1% Tween—20), and incubated for 2 h with the secondary antibody at a concentration of 1:10,000. The samples were tested with Millipore ECL (WBKLS0100, Millipore). An antibody against actin was used to control protein loading and transfer. ImageJ version 6 (National Institutes of Health) was used to measure the strip density.

### Quantitative real-time PCR

The cells were treated with or without GluOC at various doses (40,100, and 160 ng/mL). Total RNA was extracted using an RNA Simple Total RNA Kit (cat. no. DP419, Tiangen Biotech, Co., Ltd.). The purity and quality of RNA were examined by a NanoDrop2000 (Thermo Scientific, Wilmington, DE). A total of 1 µg of RNA was reverse transcribed into cDNA using the TransScript One-Step gDNA Removal and cDNA Synthesis SuperMix (cat. no. A T311‑03, TransGen Biotech.). Then, quantitative real-time PCR (qRT–PCR) was performed using the SYBR Green qPCR kit (cat. no. AQ132‑24, TransGen Biotech.). The thermal cycle conditions were 94° 30 s, followed by 40 cycles of 94° for 5 s, 55° for 15 s, and 72° for 10 s. Quantification was based on the 2^(-△△Ct) method. β-Actin served as a control, and the primer sequences are shown in Table 1.

**Table 1:** Primers for qRT–PCR

Gene	Primer sequences (5’ → 3’)
MMP13	F: AAATTATGGAGGAGATGCCCATT R: TCCTTGGAGTGGTCAAGACCTAA
IL8	F: TTCACTGGCATCTTCACTGATTCTT R: CTGCGCCAACACAGAAATTAT
VEGF	F: CAGAATCATCACGAAGTG R: TCTGCATGGTGATGTTGGAC
PTHrP	F: CTGGTTCAGCAGTGGAGC R: TTCTGCGATCAGATGGTG
β-Actin	F: AGATGTGGTCAGCAAGCAG R: GCGCAAGTTAGGTTTTGTCA

### Statistical analysis

Data are presented as the mean ± standard deviation of at least three independent experiments. The significance of differences between different groups was analyzed by one-way ANOVA with Tukey’s post-hoc test of variance using GraphPad Prism software (version 6.0, GraphPad software, Inc.). P < 0.05 was considered statistically significant.

## Results

### GluOC promotes the proliferation of MDA-MB-231 cells

**Fig. 1:**
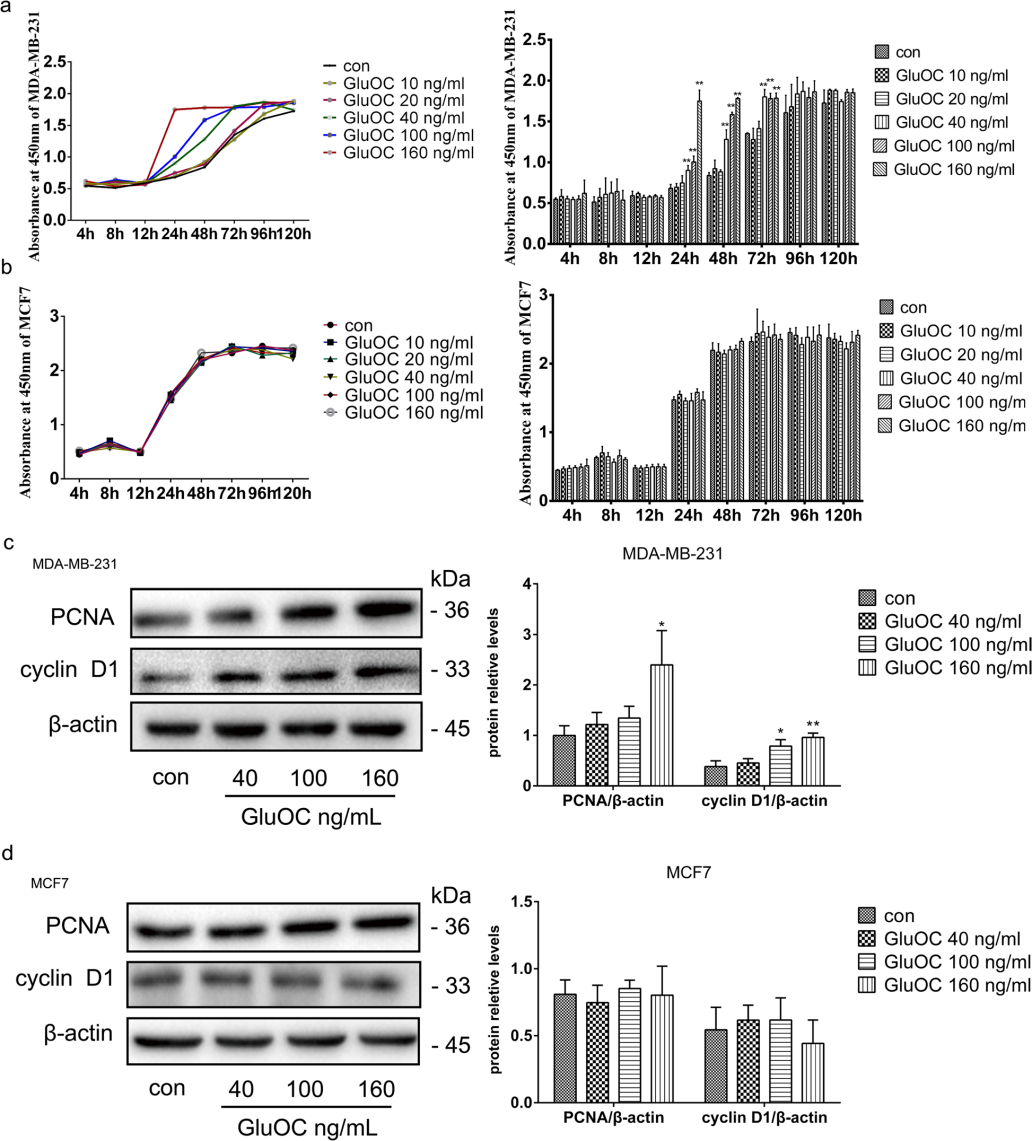
GluOC promotes MDA-MB-231 cell proliferation. **a** Growth curves of MDA-MB-231 cells treated with different doses of GluOC (10, 20, 40, 100, and 160 ng/ml) for different times (8, 12, 24, 48, 72, 96, and 120 h). **b** Growth curves of MCF7 cells treated with various doses of GluOC (10, 20, 40, 100 and 160 ng/ml) for different times (8, 12, 24, 48, 72, 96, and 120 h). **c** The protein levels of PCNA and cyclin D1 in MDA-MB-231 cells before and after GluOC stimulation were determined by western blotting and quantified densitometrically with ImageJ software. β-Actin was used as the control. **d** The protein levels of PCNA and cyclin D1 in MCF7 cells before and after GluOC stimulation were detected by western blotting. The results represent at least three independent experiments, and the data are presented as the mean ± SD (*n* = 3). *P < 0.05 and **P < 0.01, vs. the control group (con = control)

To clarify the effect of GluOC on the proliferation of breast cancer cells, MDA-MB-231 and MCF7 cells were treated with different doses of GluOC (10, 20, 40, 100, and 160 ng/mL) for different times (4, 8, 12, 24, 48, 72, 96, and 120 h). After 24 h of culture with GluOC, the proliferation of MDA-MB-231 cells treated with 40, 100, and 160 ng/mL GluOC was significantly improved (Fig. 1a). The proliferation of MCF7 cells treated with GluOC showed no significant change (Fig. 1b). Based on these results, the 40, 100, and 160 ng/mL concentrations of GluOC were selected for subsequent experiments. Similarly, GluOC (100 and 160 ng/mL) increased the PCNA and cyclin D1 content in MDA-MB-231 cells compared with that in the control group (Fig. 1c). However, GluOC had no obvious effect on the PCNA and cyclin D1 protein levels in MCF7 breast cancer cells (Fig. 1d). These results showed that GluOC accelerated the proliferation of MDA-MB-231 cells but did not influence MCF7 cells.

### GluOC promotes the migration of MDA-MB-231 cells

**Fig. 2:**
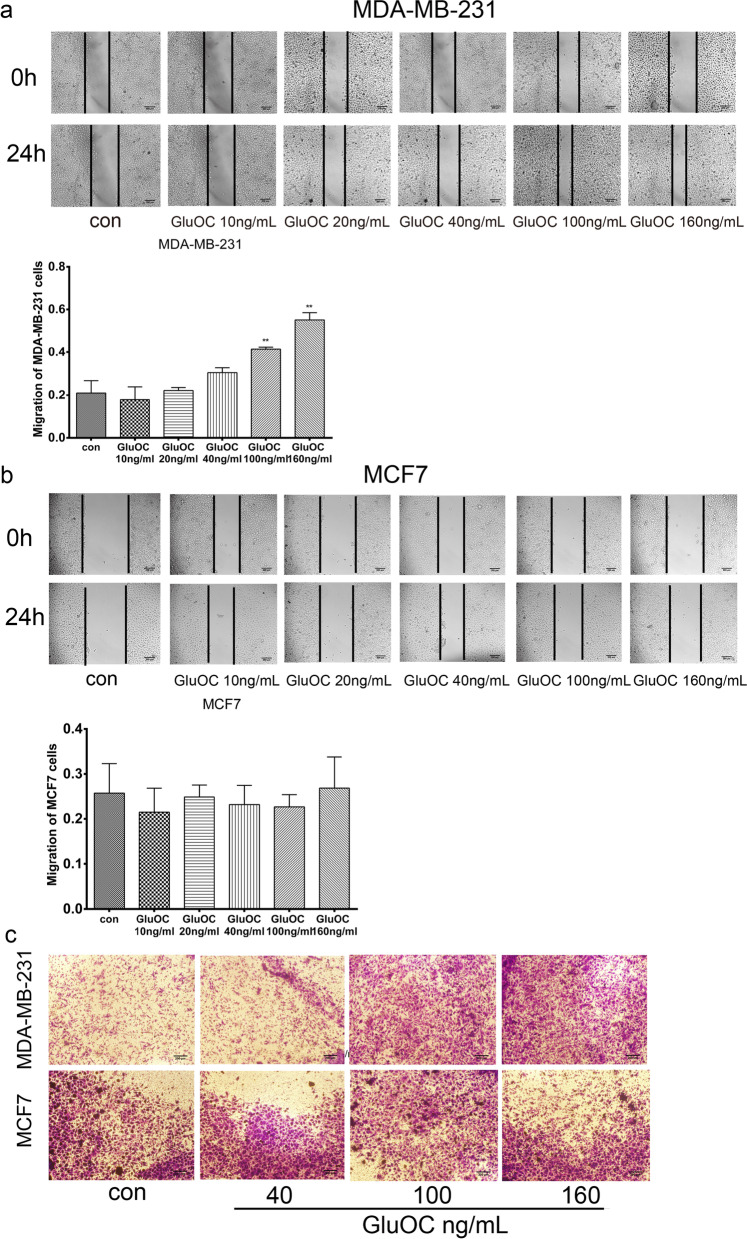
GluOC promotes MDA-MB-231 cell migration. **a** Wound healing assay of MDA-MB-231 cells. The bar chart shows the wound healing ability, magnification × 100. **b** Wound healing assay of MCF7 breast cancer cells. The bar chart shows the wound healing ability, magnification × 100. The results represent at least three independent experiments, and the data are presented as the mean ± SD (*n* = 3). **c** Transwell assay. MDA-MB-231 and MCF-7 cells were transferred onto the upper membrane of Transwell plates. Photographs were taken with a microscope for counting, with at least five fields photographed per treatment group, magnification × 100. *P < 0.05 and **P < 0.01, vs. the control group (con = control)

To determine whether GluOC might facilitate the migration of breast cancer cells, wound healing and transwell experiments were performed on MDA-MB-231 and MCF7 cells. The wound healing assay results are shown in Fig. 2a & b. After culture for 24 h with GluOC, the wound healing of MDA-MB-231 breast cancer cells was markedly increased compared with that of the control group, but migration was not obviously changed in MCF7 cells. The transwell assay results (Fig. 2c) showed that the GluOC treatment (100 and160 ng/mL) increased the capacity of MDA-MB-231 cells to penetrate the cellulose membrane in a manner depending on the GluOC concentration, while GluOC had no obvious effect on the migration of MCF7 cells. These results suggested that GluOC promotes the migration of MDA-MB-231 cells, but does not significantly influence the migration of MCF7 cells.

### GluOC promotes the expression of MMP2, MMP13 and VEGF in MDA-MB-231 cells

**Fig. 3:**
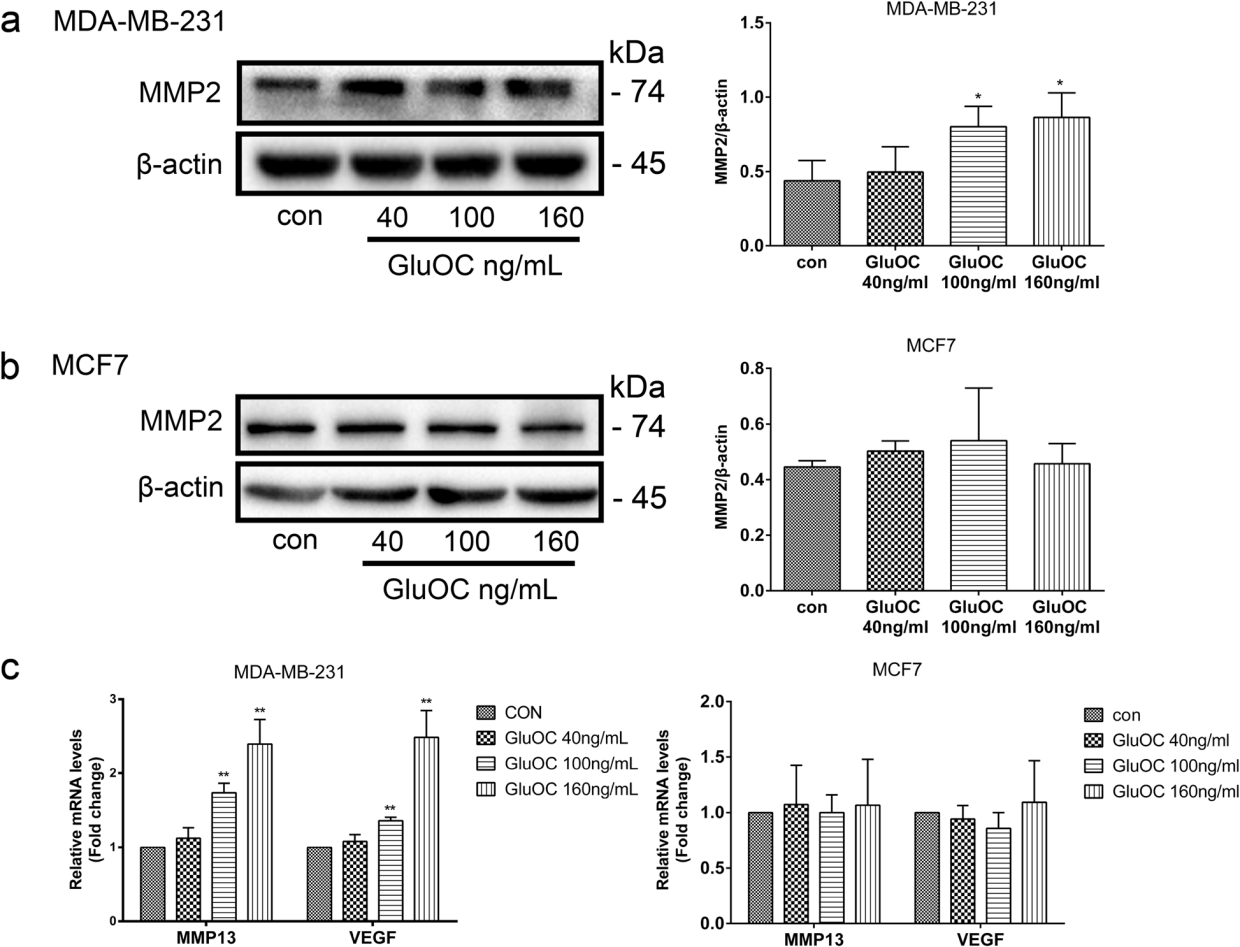
GluOC promotes the expression of MMP2, MMP13 and VEGF in MDA-MB-231 cells. **a** The expression of MMP2 in MDA-MB-231 cells was analyzed by western blotting and quantified densitometrically with ImageJ software. **b** Western blot analysis of MMP2 expression in MCF7 cells. **c** mRNA expression levels of MMP13 and VEGF in MDA-MB-231 and MCF7 cells. The results represent at least three independent experiments, and the data are presented as the mean ± SD (*n* = 3). *P < 0.05 and **P < 0.01 vs. the control group (con = control). MMP2, matrix metalloproteinase 2; MMP13, matrix metalloproteinase 13; VEGF, vascular endothelial growth factor

Matrix metalloproteinases (MMPs) and vascular endothelial growth factor (VEGF) play vital roles in cancer migration, invasion and metastasis [27, 28]. To study the impact of GluOC on the degradation of extracellular matrix and induction of angiogenesis, the expression of MMPs and VEGF was analyzed. After GluOC treatment, the content of MMP2 protein in MDA-MB-231 cells increased significantly (Fig. 3a). GluOC increased the mRNA content of MMP13 and VEGF in MDA-MB-231 cells, while it did not obviously affect the expression levels of MMP2, MMP13 and VEGF in MCF7 cells (Fig. 3b & c). All the above results further confirmed that GluOC stimulates the migration of MDA-MB-231 cells.

### GluOC promotes the EMT process in MDA-MB-231 cells

**Fig. 4:**
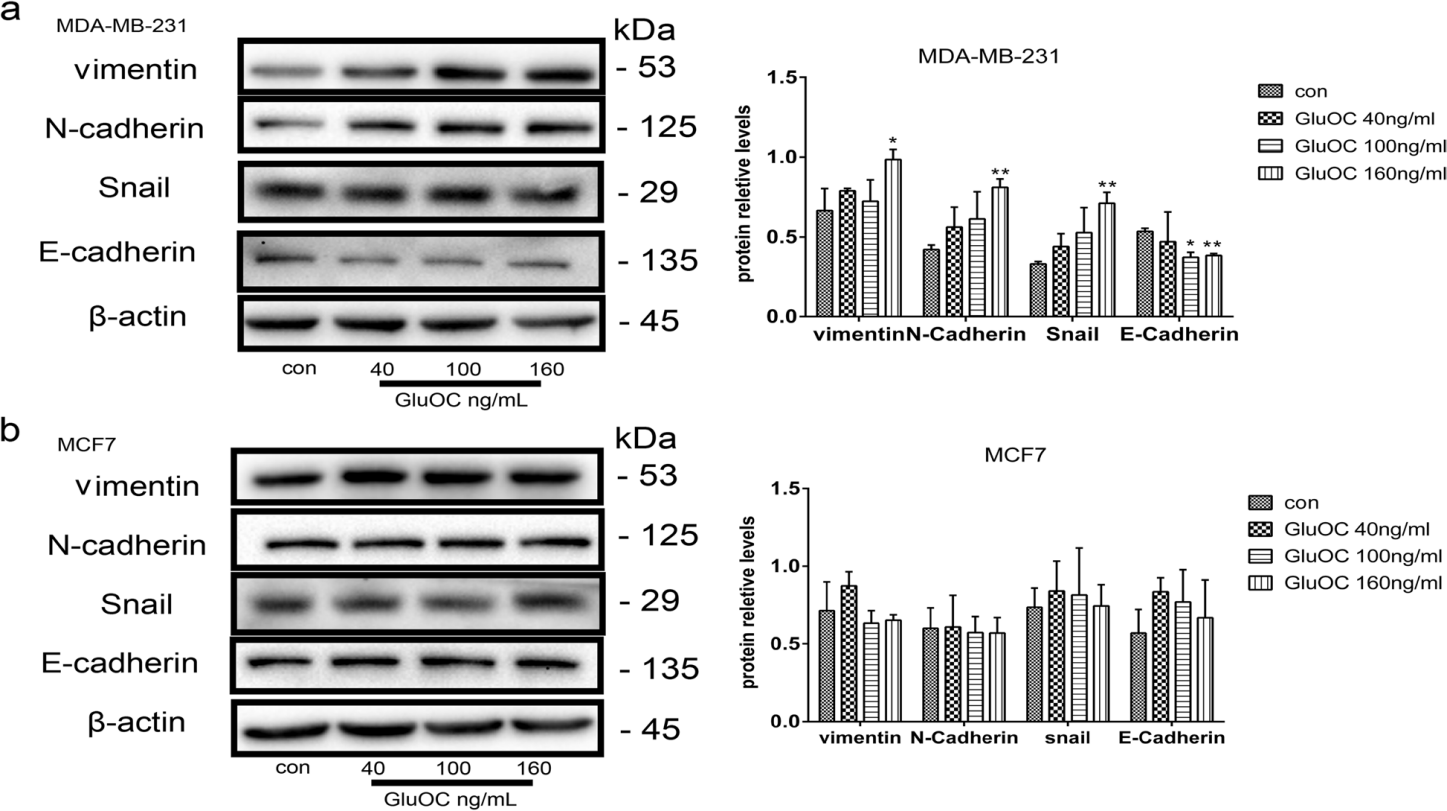
GluOC promotes EMT and metastasis of MDA-MB-231 cells. **a** Western blotting was performed to illustrate the influence of various doses of GluOC on the protein levels of vimentin, Snail, N-cadherin and E-cadherin in MDA-MB-231 cells. **b** Western blotting was used to detect the effect of different doses of GluOC on the protein levels of vimentin, Snail, N-cadherin and E-cadherin in MCF7 breast cancer cells. β-Actin was used as an internal reference. The expression level was quantified densitometrically with the ImageJ software. The results represent at least three independent experiments, and the data are presented as the mean ± SD (*n* = 3). *P < 0.05 and **P < 0.01, vs. the control group (con = control)

EMT is a pivotal process promoting cancer cell metastasis [29]. We explored the function of GluOC in the EMT of breast cancer cells by assessing the levels of vimentin, N-cadherin, Snail and E-cadherin. Our results showed that vimentin, Snail and N-cadherin protein levels in MDA-MB-231 cells treated with GluOC were significantly increased, while the E-cadherin levels were decreased (Fig. 4a). In MCF7 cells, GluOC treatment did not obviously affect the levels of vimentin, Snail, N-cadherin and E-cadherin (Fig. 4b). In summary, our study illustrated that GluOC promotes the EMT of MDA-MB-231 cells.

### GluOC promotes the expression of PTHrP and IL-8 in MDA-MB-231 cells

**Fig. 5:**
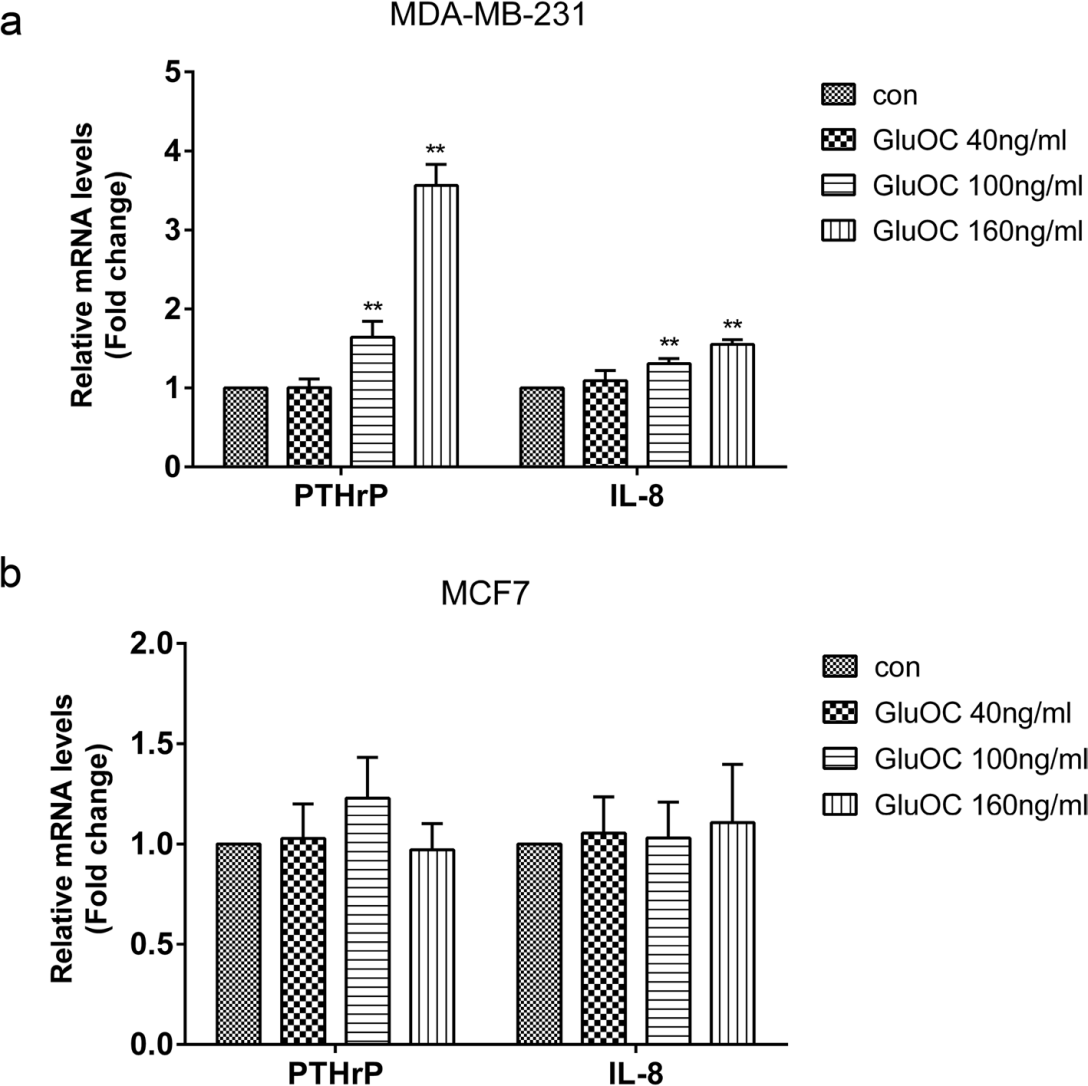
GluOC increases the levels of PTHrP and IL-8 in MDA-MB-231 cells. **a** qRT–PCR was used to detect the PTHrP and IL-8 gene levels in MDA-MB-231 cells. **b** qRT–PCR was used to detect the levels of PTHrP and IL-8 in MCF7 cells. The results represent at least three independent experiments, and the data are presented as the mean ± SD (*n* = 3). *P < 0.05 and **P < 0.01, vs. the control group (con = control)

Bone metastasis of breast cancer often causes osteolytic damage. Both IL-8 and PTHrP can act as osteolytic factors in breast cancer cells [30]. Therefore, we studied the effect of GluOC on the levels of PTHrP and IL-8. We found that after GluOC treatment, the expression of the PTHrP and IL-8 genes in MDA-MB-231 cells was increased (Fig. 5a). In MCF7 cells, the PTHrP and IL-8 gene expression levels were not obviously changed (Fig. 5b). These results showed that GluOC facilitates the osteolytic potency of MDA-MB-231 cells.

### GluOC promotes the TGF-β/ SMAD3 signaling pathway

**Fig. 6:**
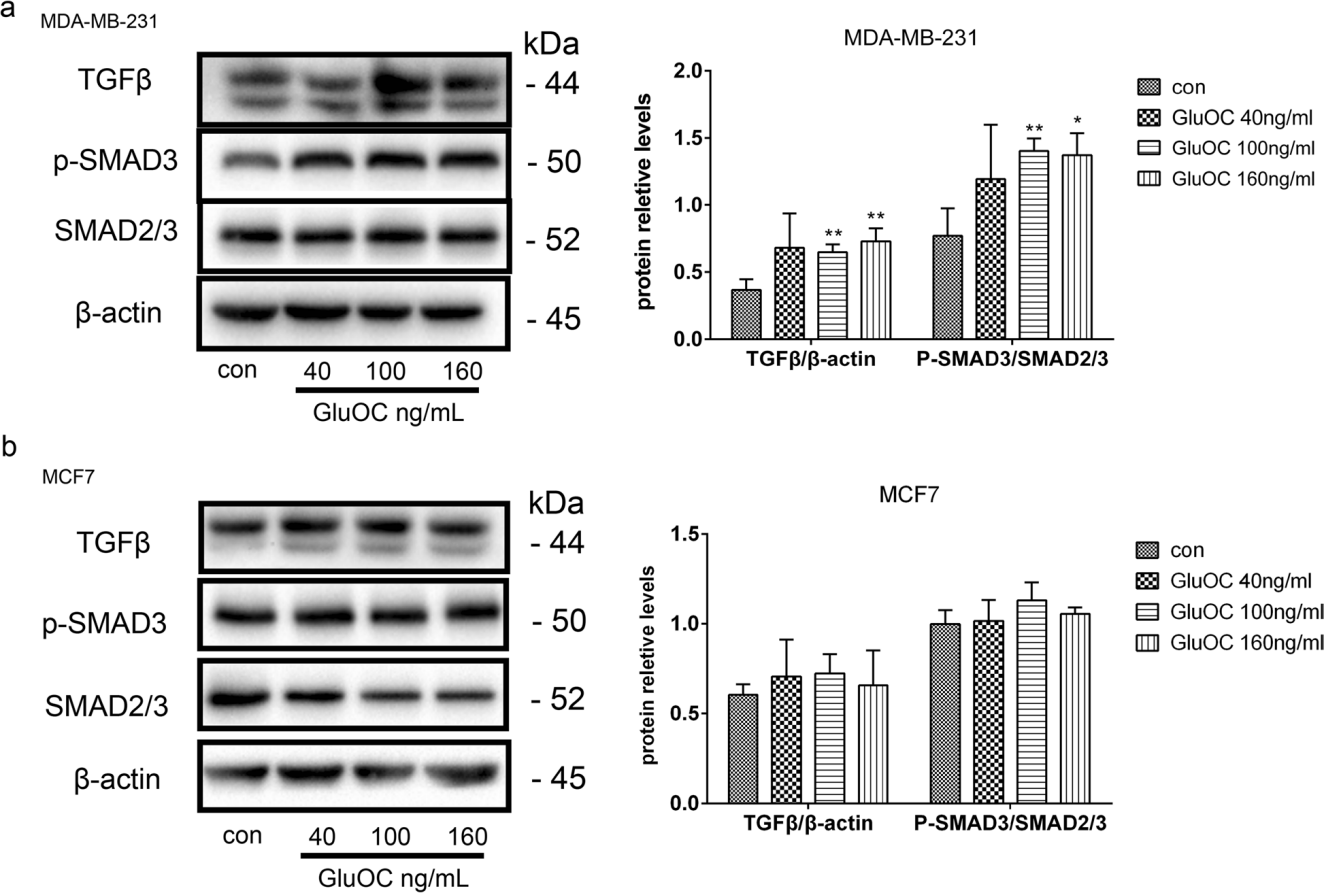
GluOC promotes the expression of TGF-β and p-SMAD3 in MDA-MB-231 cells. **a** Western blotting was used to analyze the levels of TGF-β, SMAD2/3, p-SMAD3 and β-actin in MDA-MB-231 cells. **b** Western blotting was used to analyze the levels of TGF-β, SMAD2/3, p-SMAD3 and β-actin in MCF7 breast cancer cells. The results represent at least three independent experiments, and the data are presented as the mean ± SD (*n* = 3). *P < 0.05 and **P < 0.01, vs. the control group (con = control)

The TGF-β/SMAD3 pathway plays an indispensable role in tumor development [31]. Therefore, the effect of GluOC on the TGF-β/ SMAD3 pathway in MDA-MB-231 and MCF7 cells was detected by western blotting. When the GluOC concentrations were 100 ng/mL and 160 ng/mL, the total amount of SMAD2/3 in MDA-MB-231 cells was not affected, while the phosphorylation of SMAD3 was significantly increased (Fig. 6a). In addition, TGF-β protein levels after GluOC treatment were markedly increased compared with those in the control group (Fig. 6a). Conversely, in MCF7 cells, different concentrations of GluOC did not affect the phosphorylation of SMAD3, the total amount of SMAD2/3, or the protein level of TGF-β (Fig. 6b). Our study indicated that GluOC can effectively improve the expression of TGF-β/SMAD3 in MDA-MB-231 cells, while GluOC did not have effects on MCF7 breast cancer cells.

### GluOC promotes the proliferation and migration of MDA-MB-231 cells through the TGF-β/ SMAD3 pathway

**Fig. 7:**
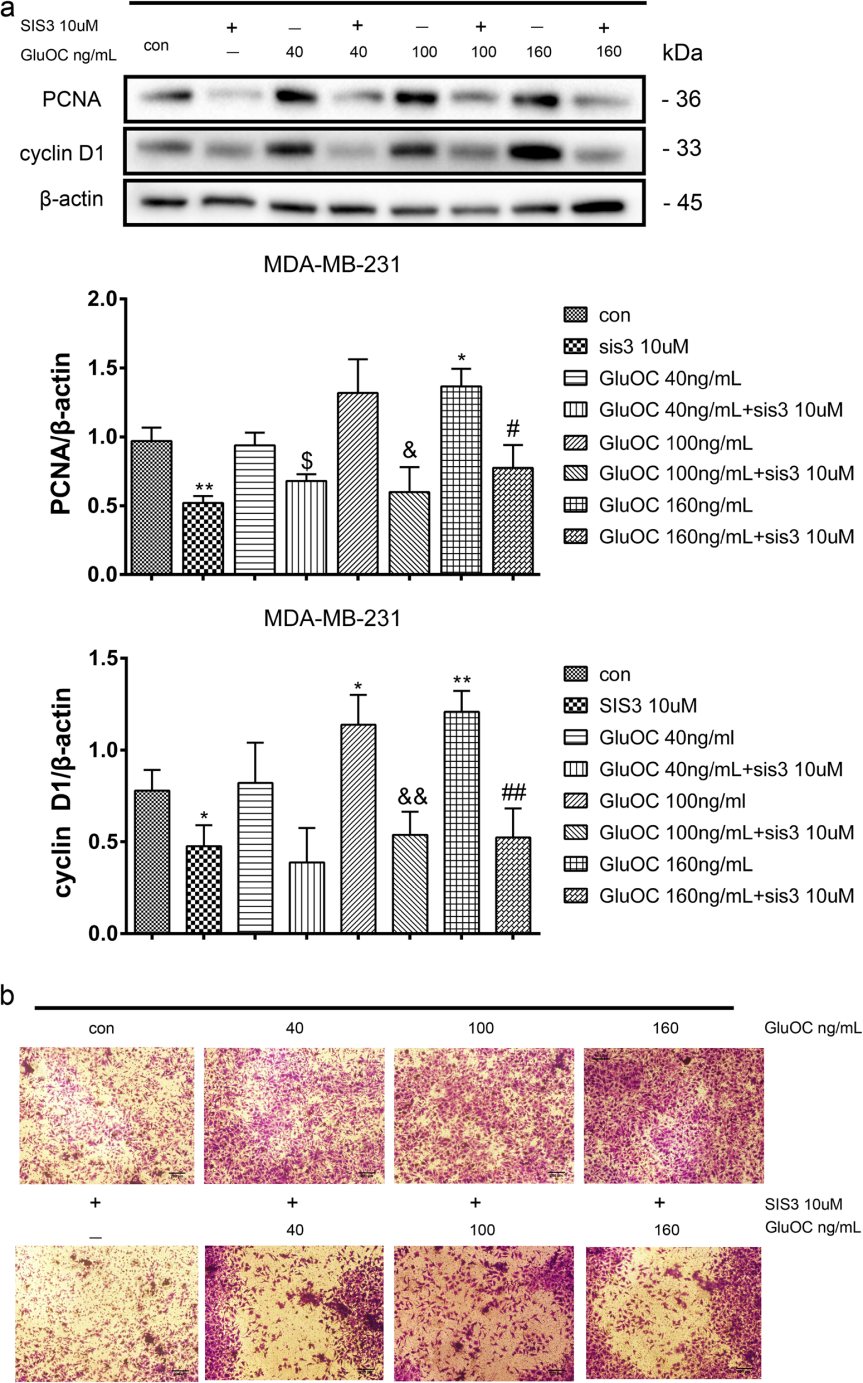
GluOC stimulates the growth and migration of MDA-MB-231 cells by activating the TGF-β/SMAD3 pathway. **a** After adding SIS3 and different concentrations of GluOC, we detected cyclin D1 and PCNA protein expression by western blotting. The results represent at least three independent experiments, and the data are presented as the mean ± SD (*n* = 3). *P < 0.05 and **P < 0.01, vs. the control group (con = control); ^$^*P* < 0.05, ^$$^*P* < 0.01 vs. the GluOC 40 ng/mL group; ^&^*P* < 0.05, ^&&^*P* < 0.01 vs. the GluOC 100 ng/mL group; ^#^*P* < 0.05, ^##^*P* < 0.01 vs. the GluOC 160 ng/mL group. **b** After adding SIS3 and different concentrations of GluOC, we tested the migration capacity of MDA-MB-231 cells by Transwell assay. Photographs were taken with a microscope, with at least five field captured per treatment group, magnification × 100

To further explore the function of SMAD3 in the promotion of the growth and migration of MDA-MB-231 cells by GluOC, the SMAD3 phosphorylation inhibitor SIS3 (10 μM) was used to treat MDA-MB-231 cells. Our western blot analysis revealed that SIS3 abrogated the GluOC-induced increase in PCNA and cyclin D1 in MDA-MB-231 cells (Fig. 7a). Additionally, the capacity of MDA-MB-231 cells to cross the cellulose membrane was reduced after the addition of SIS3 (Fig. 7b). These findings suggested that SIS3 inhibits the increased growth and migration of MDA-MB-231 cells induced by GluOC.

### GluOC increases MMP2, MMP13 and VEGF in MDA-MB-231 cells through the TGF-β/SMAD3 pathway

**Fig. 8:**
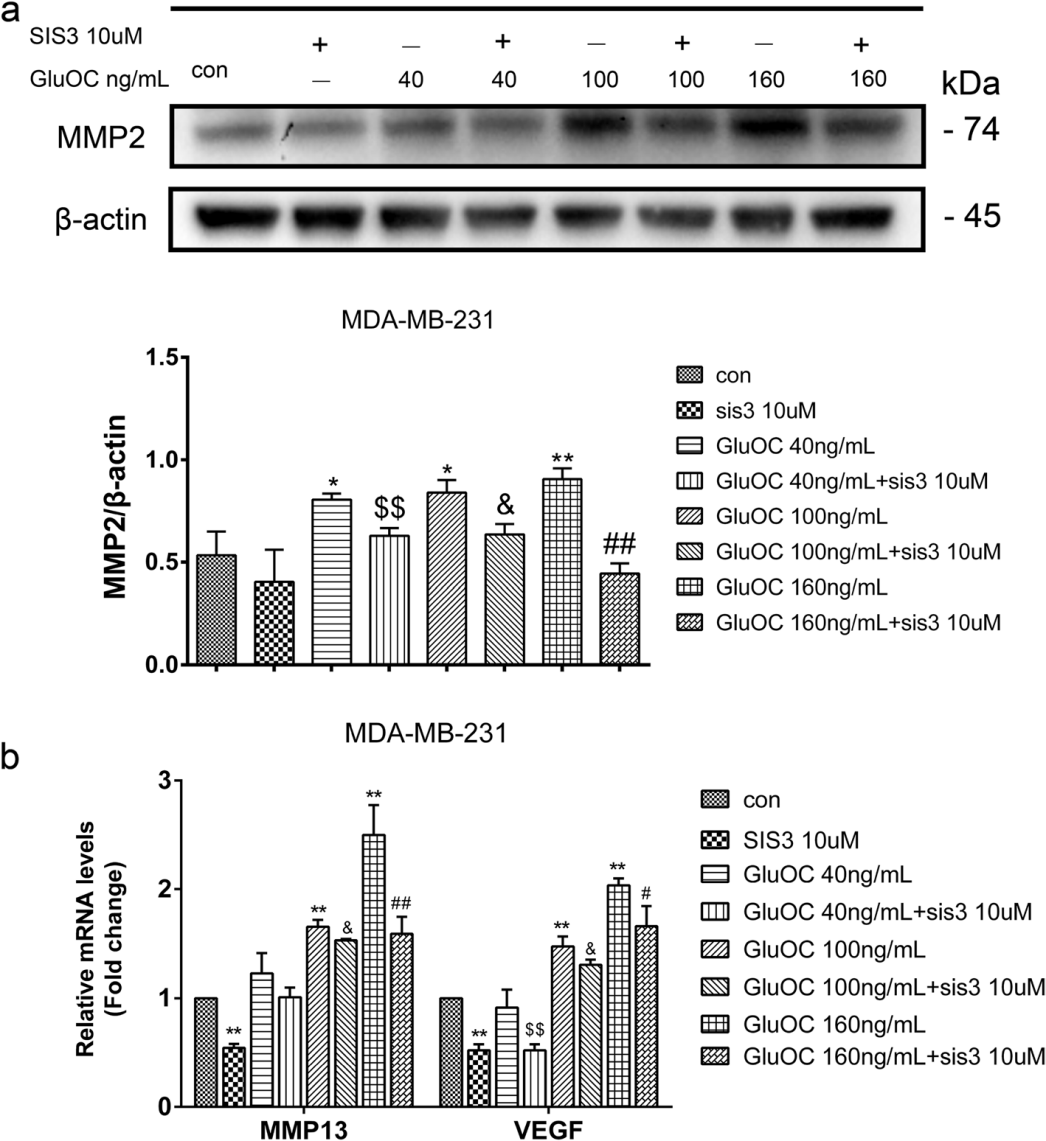
GluOC induces the expression of MMP2, MMP13 and VEGF in MDA-MB-231 cells by promoting phosphorylation of SMAD3. **a** Western blotting was used to detect the content of MMP2. The protein expression level was quantified densitometrically with ImageJ software. **b** GluOC promoted the expression of MMP13 and VEGF in MDA-MB-231 cells via the TGF-β/SMAD3 pathway. The results represent at least three independent experiments, and the data are presented as the mean ± SD (*n* = 3). *P < 0.05 and **P < 0.01, vs. the control group (con = control); ^$^*P* < 0.05, ^$$^*P* < 0.01 vs. the GluOC 40 ng/mL group; ^&^*P* < 0.05, ^&&^*P* < 0.01 vs. the GluOC 100 ng/mL group; ^#^*P* < 0.05, ^##^*P* < 0.01 vs. the GluOC 160 ng/mL group

Subsequently, the role of GluOC in promoting cancer cell migration via SMAD3 was further verified. After adding SIS3, the increased expression of MMP2 induced by GluOC was suppressed by SIS3. Additionally, SIS3 reversed the increase in the gene expression of MMP13 and VEGF induced by GluOC (Fig. 8a & b). These results further verified that GluOC increased the transfer of MDA-MB-231 cells via the TGF-β/SMAD3 pathway.

### GluOC promotes EMT of MDA-MB-231 cells via phosphorylation of SMAD3

**Fig. 9:**
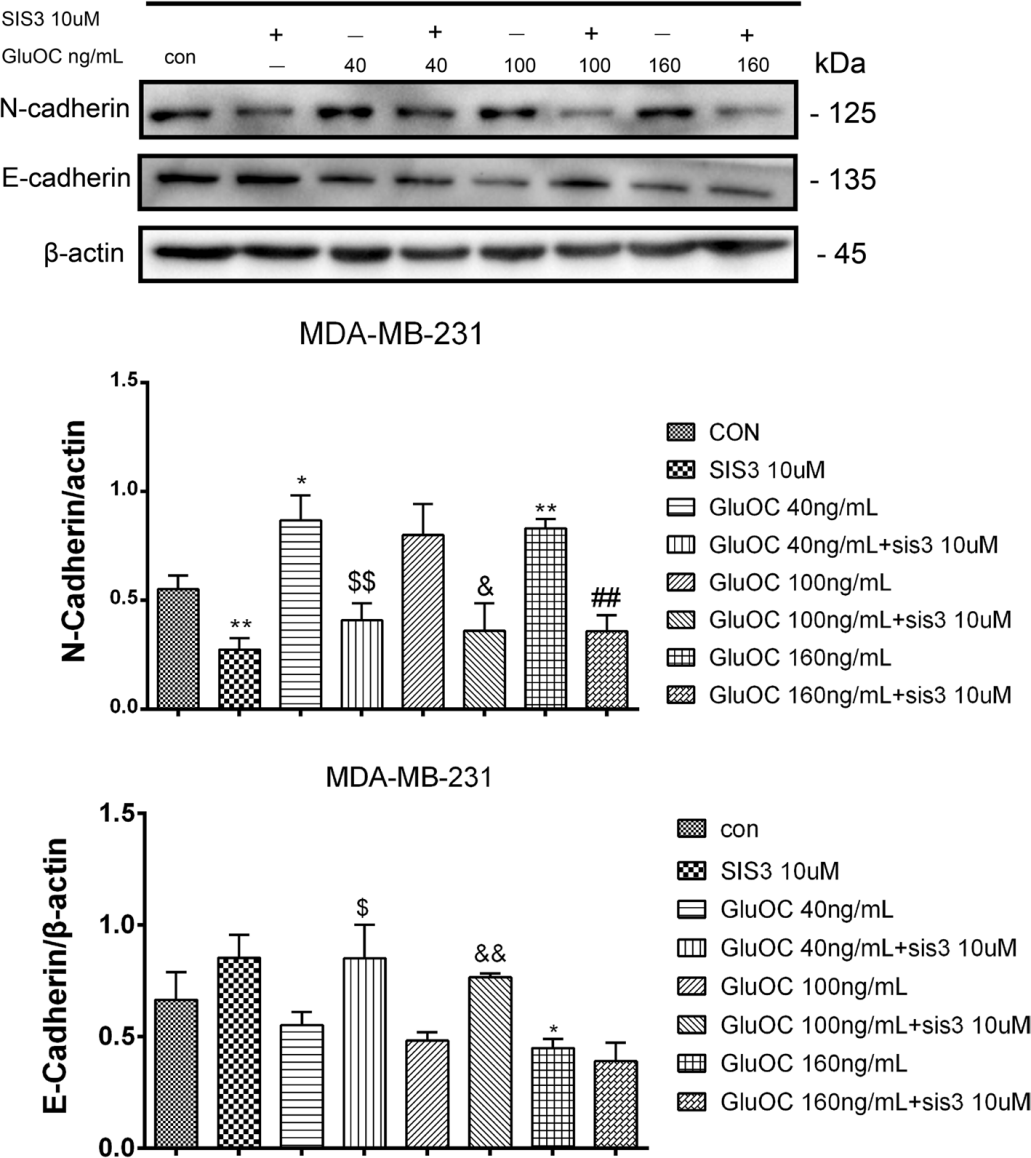
GluOC promotes EMT of MDA-MB-231 cells via the TGF-β/SMAD3 pathway. Western blotting was used to analyze the levels of N-cadherin and E-cadherin. The results represent at least three independent experiments, and the data are presented as the mean ± SD (*n* = 3). *P < 0.05 and **P < 0.01, vs. the control group (con = control); ^$^*P* < 0.05, ^$$^*P* < 0.01 vs. the GluOC 40 ng/mL group; ^&^*P* < 0.05, ^&&^*P* < 0.01 vs. the GluOC 100 ng/mL group; ^#^*P* < 0.05, ^##^*P* < 0.01 vs. the GluOC 160 ng/mL group

To further confirm whether GluOC stimulates EMT of MDA-MB-231 cells through the TGF-β/SMAD3 pathway, western blotting was used to detect related protein expression. The western blot results showed that SIS3 abrogated the GluOC-induced increase in the N-cadherin protein. Additionally, it was demonstrated that the addition of SIS3 reversed the inhibitory effects of GluOC on the E-cadherin protein (Fig. 9). These findings suggested that SIS3 inhibits the phosphorylation of SMAD3 and blocks the promoting effect of GluOC on MDA-MB-231 cell EMT.

### GluOC promotes the expression of PTHrP and IL-8 in MDA-MB-231 cells via phosphorylation of SMAD3

**Fig. 10:**
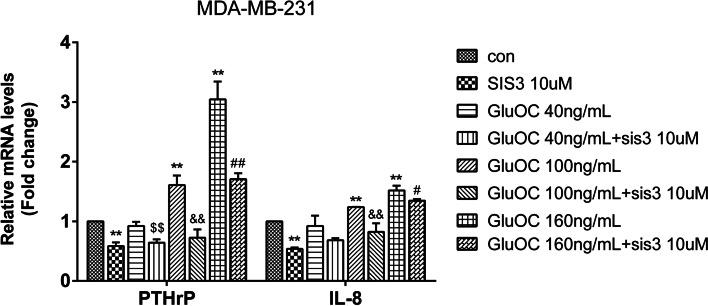
GluOC promotes the expression of PTHrP and IL-8 in MDA-MB-231 cells by activating the TGF-β/SMAD3 pathway. After SIS3 and GluOC were added, the expression of the PTHrP and IL-8 genes was detected by qRT–PCR. The results represent at least three independent experiments, and the data are presented as the mean ± SD (*n* = 3). *P < 0.05 and **P < 0.01, vs. the control group (con = control); ^$^*P* < 0.05, ^$$^*P* < 0.01 vs. the GluOC 40 ng/mL group; ^&^*P* < 0.05, ^&&^*P* < 0.01 vs. the GluOC 100 ng/mL group; ^#^*P* < 0.05, ^##^*P* < 0.01 vs. the GluOC 160 ng/mL group. IL8: GluOC, uncarboxylated osteocalcin; IL-8: interleukin-8; PTHrP: parathyroid hormone related protein

We next explored whether GluOC in MDA-MB-231 cells induces osteolytic destruction via the TGF-β/SMAD3 signaling pathway. The results showed that GluOC increased the mRNA expression of PTHrP and IL-8 in cells. However, the addition of SIS3 attenuated this effect and reversed the increase in PTHrP and IL-8 induced by GluOC (Fig. 10). The study showed that GluOC increases the osteolytic properties of MDA-MB-231 cells by activating the TGF-β/SMAD3 signaling pathway.

## Discussion

**Fig. 11:**
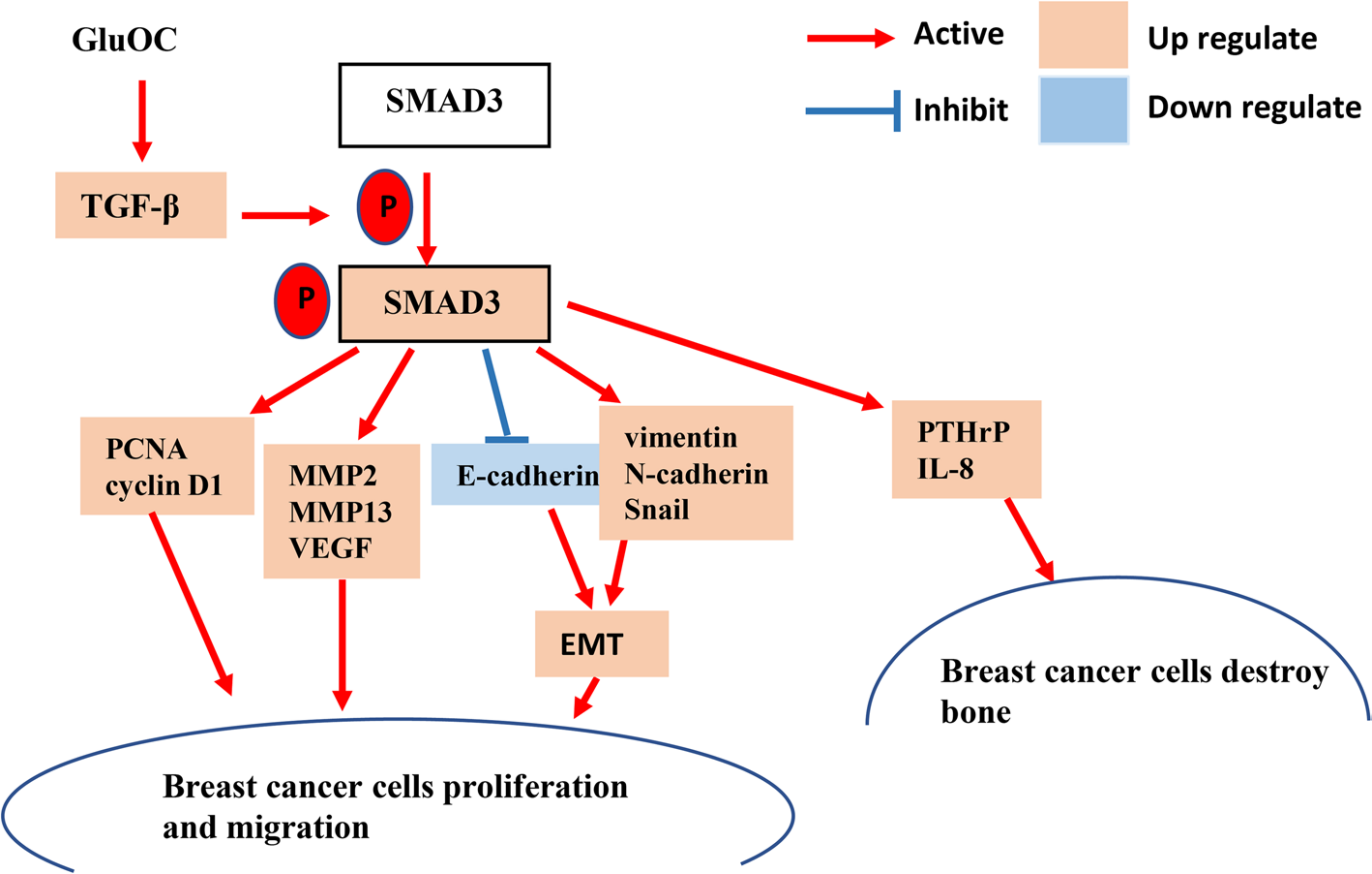
Model of GluOC functions in the growth and migration of MDA-MB-231 cells. By stimulating the TGF-β/SMAD3 pathway, GluOC accelerates the growth and migration of MDA-MB-231 cells

According to literature reports, serum osteocalcin levels in patients with breast cancer and breast cancer bone metastasis are significantly increased [24]. However, the mode of action is still unclear. Our study shows that GluOC facilitates the growth and migration of MDA-MB-231 cells through the TGF-β/SMAD3 pathway and promotes the expression of the PTHrP and IL8 genes, which suggests that GluOC may be related to TNBC osteolytic bone metastasis. (Fig. 11).

Breast cancer is the leading cause of death in females worldwide, and metastasis leads to a high mortality rate [32, 33]. Cancer tends to spread to the bone, and the most common form of metastasis in breast cancer is osteolytic metastasis [34]. TNBC is highly metastatic and is characterized by ER, PR and HER2 deficiency. TNBC accounts for nearly 20% of all breast cancers, carries a death rate of 83%, and is on the rise globally. TNBC is the most challenging subtype to treat due to its lack of a specific target [35]. MDA-MB-231 is a TNBC cell line that can cause lytic bone destruction [36]. MCF-7 is a noninvasive cell line that is ER-positive and normally characterized by low metastatic potential [37]. Therefore, in this study, MDA-MB-231 and MCF7 cells were employed.

Osteocalcin is a small noncollagen protein that is mainly produced by osteoblasts and plays a role in regulating glucose and lipid metabolism [38]. Recent research has found that GluOC is related to the occurrence and progression of cancer [20]. GluOC has been found to facilitate the growth and migration of lung tumors through the aggregation of neutrophils [39]. GluOC has also been found to induce the growth of prostate cancer cells through GPRC6A [40]. Studies have demonstrated that osteocalcin has a tumor-promoting effect in pancreatic ductal carcinoma and osteosarcoma [22, 23]. An increasing number of studies have confirmed that serum osteocalcin levels are significantly increased in breast cancer patients with bone metastasis [41, 42]. However, whether GluOC has an effect on breast cancer cells remains unclear. This research showed that GluOC promotes the viability and growth of MDA-MB-231cells. GluOC enhanced the viability of MDA-MB-231 cells and increased the levels of PCNA and cyclin D1. Our results suggest that GluOC can induce the proliferation of MDA-MB-231 cells but has no obvious influence on MCF7 cells.

The metastasis of breast cancer cells involves multiple steps: growth in situ, degradation of extracellular matrix, EMT, angiogenesis, and subsequent migration into the blood circulation to reach target organs. MMPs are zinc-dependent neutral endopeptidases that can selectively degrade ECM and are related to cancer invasion and metastasis [43]. MMP2 is elevated in many tumors, and its increase promotes the growth and migration of malignant cancer cells [44]. In breast cancer, increased MMP13 is related to bone metastasis [30]. Angiogenesis is another necessary factor for cancer metastasis. VEGF is responsible for regulating angiogenesis, and angiogenesis is a determinant of growth and metastasis in breast cancer [45]. The impact of GluOC on the metastasis of MDA-MB-231 and MCF7 cells was investigated. The results showed that GluOC improved the wound healing ability and ability to penetrate a cellulose membrane of MDA-MB-231 cells but had no obvious effect on MCF7 cells. Then, we detected the expression of MMP2 protein by western blot. In concordance with the cell migration results, GluOC promoted the expression of MMP2 in MDA-MB-231 cells but did not induce the expression of MMP2 in MCF7 cells. Subsequently, we detected the gene expression levels of MMP13 and VEGF by qRT–PCR. The results showed that GluOC enhanced the expression of the MMP13 and VEGF genes in MDA-MB-231 cells but did not influence them in MCF7 cells. Our results indicate that GluOC strengthens the migration ability of MDA-MB-231 cells but does not significantly affect MCF7 breast cancer cells.

During metastasis, breast cancer cells undergo EMT, which promotes invasion of surrounding tissues by cancer cells from the primary tumor site. A decrease in N-cadherin, vimentin and Snail expression and an increase in E-cadherin protein content promote the EMT process and increase the invasion and metastasis of cancer cells [46]. We also found that after adding GluOC, the N-cadherin, vimentin and Snail protein levels in MDA-MB-231 cells were significantly increased, and the E-cadherin level was significantly decreased. In summary, GluOC promotes EMT of MDA-MB-231 cells but does not significantly affect MCF7 breast cancer cells.

Osteolytic metastasis is the most common form of breast tumor metastasis. Breast tumors generate molecules that activate osteoclast bone absorption when they invade the microenvironment of bone tissue [47]. PTHrP is highly increased in breast cancer cells and is related to the growth and metastasis of cancer cells [48]. PTHrP also stimulates the differentiation and activation of osteoclasts [49]. IL-8 is increased in diverse cancer cells with different metastatic potentials and is related to the enhancement of breast cancer cell metastasis to bone [50]. Moreover, IL-8 facilitates osteoclastogenesis and injury of bone in metastatic bone diseases [51]. Based on these previous findings, the mRNA expression of PTHrP and IL-8 in MDA-MB-231 and MCF7 cells was detected. Our study showed that GluOC significantly increased the amount of PTHrP and IL-8 in MDA-MB-231 cells, but the content of PTHrP and IL-8 in MCF7 cells did not change significantly. This result suggests that GluOC may be related to the osteolytic properties of MDA-MB-231 cells.

Activation of diverse signaling pathways leads to breast cancer cell proliferation and metastasis. TGF-β is a secreted cytokine that can regulate tumor cell proliferation and migration. The TGF-β signaling network induces the key effector molecules SMAD2, SMAD3, and SMAD4. SMAD3, as an intermediate in the typical TGF-β signaling pathway, has an indispensable influence on TGF-β mediated transcriptional regulation [52]. SMAD3 can also regulate N-cadherin, E-cadherin, vimentin and Snail [53], thus affecting the EMT process. SMAD3 can also affect the proliferation and metastasis of cancer cells [54]. The results of Lian GY et al. showed that TGF-β can increase MMP2, MMP13, and VEGF to promote tumor invasion and metastasis [55–57]. There is also evidence that TGF-β/SMAD3 can regulate IL-8 and PTHrP, which can stimulate bone metastasis of breast cancer cells [58, 59]. Our results showed that GluOC stimulates TGF-β expression and SMAD3 phosphorylation in MDA-MB-231 cells. After adding SIS3, the increased proliferation of MDA-MB-231 cells induced by GluOC was abrogated. It appears that SIS3 blocks GluOC's promotion of the cyclin D1 and PCNA proteins. SIS3 also blocked the migration of MDA-MB-231 cells. First, SIS3 inhibited the promotion of the expression of MMP2, MMP13 and VEGF induced by GluOC in tumor cells. Second, SIS3 eliminated the increase in N-cadherin and the decrease in E-cadherin induced by GluOC in MDA-MB-231 cells. This result indicates that SIS3 weakened the promotion of MDA-MB-231 cell EMT induced by GluOC. Finally, the addition of SIS3 attenuated the increase in PTHrP and IL-8 mRNA expression induced by GluOC. GluOC may affect the osteolytic process of MDA-MB-231 cells via the TGF-β/SMAD3 pathway. These results further confirm that GluOC stimulates the growth and migration of MDA-MB-231 cells via the TGF-β/SMAD3 pathway.

## Conclusions

In conclusion, our research clarified for the first time that GluOC induces the proliferation and metastasis of MDA-MB-231 cells through the TGF-β/SMAD3 signaling pathway. In addition, GluOC also stimulated the expression of the IL-8 and PTHrP genes in MDA-MB-231 breast cancer cells, both of which can act as osteolytic factors. The results indicate that GluOC may be a novel therapeutic target that can be used to prevent or treat TNBC bone metastasis in the clinic. All the results of the present study are from cell-level experiments. As such, the effect of GluOC in animals is still unclear, and further experimental verification in vivo is needed.

## Supplementary Information

**Additional file 1.**

## Data Availability

All analyses and results we obtained from the experiments are included in this article.

## Abbreviations

GluOC: Uncarboxylated osteocalcin
BM: Bone metastasis
SRE: Skeletal -related events
PCNA: Proliferating cell nuclear antigen
TNBC: Triple-negative breast cancer
ER: Estrogen receptor
PR: Progesterone receptor
HER2: Human epidermal growth factor receptor 2
MMPs: Matrix metalloproteinases
MMP2: Matrix metallopeptidase 2
MMP13: Matrix metalloproteinase13
IL8: Interleukin-8
VEGF: Vascular endothelial growth factor
PTHrP: Parathyroid hormone related protein
ECM: Extracellular matrix
