# Supplementary material for: Uncarboxylated osteocalcin promotes proliferation and metastasis of MDA-MB-231 cells through TGF-β/SMAD3 signaling pathway
Source: BMC Mol Cell Biol. 2022 Apr 12;23:18. doi: 10.1186/s12860-022-00416-7 (PMC9003967; doi:10.1186/s12860-022-00416-7)
Supplement: Supplementary file 1 — Additional file 1. [file 12860_2022_416_MOESM1_ESM.docx]

**Uncarboxylated osteocalcin promotes proliferation and metastasis of MDA-MB-231 cells through TGF-β/SMAD3 signaling pathway**

Jiaojiao Xu^1^, Luyao Ma^2^, Danqing Wang^1^, and Jianhong Yang^1*^

^1^Medical School, University of Chinese Academy of Sciences, Beijing 100049, P.R. China

^2^School of Chinese Materia Medica, Guangzhou University of Chinese Medicine, Guangzhou 510000, P.R.China

*Correspondence to: Professor Jianhong Yang, Medical School, University of Chinese Academy of Sciences, 19A Yuquan Road, Beijing 100049, P.R. China

E-mail: [yangjh@ucas.edu.cn](mailto:yangjh@ucas.edu.cn)


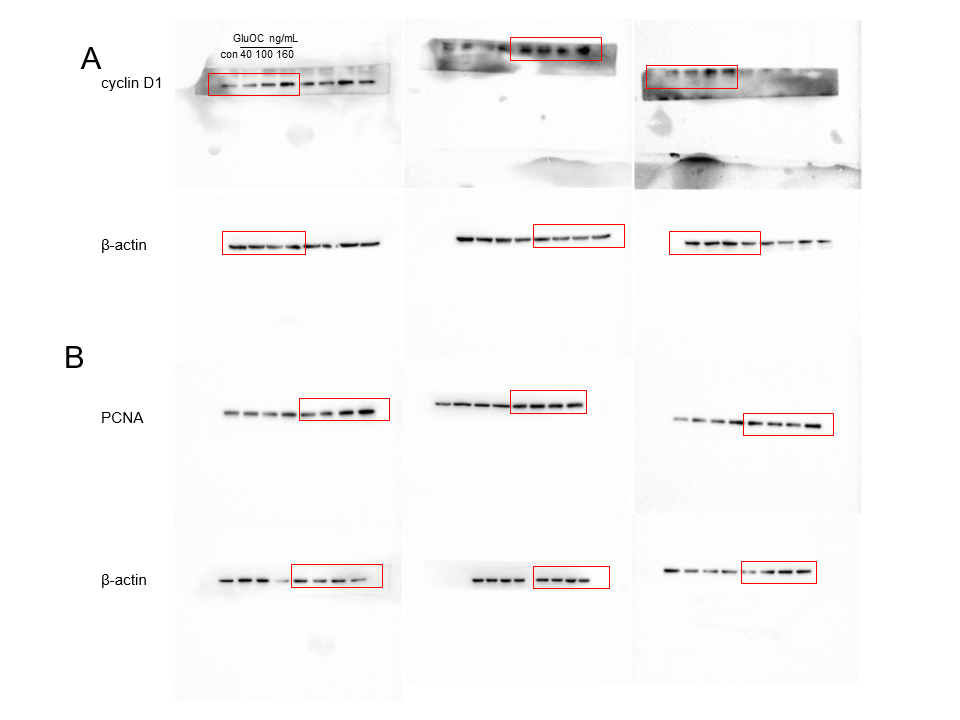


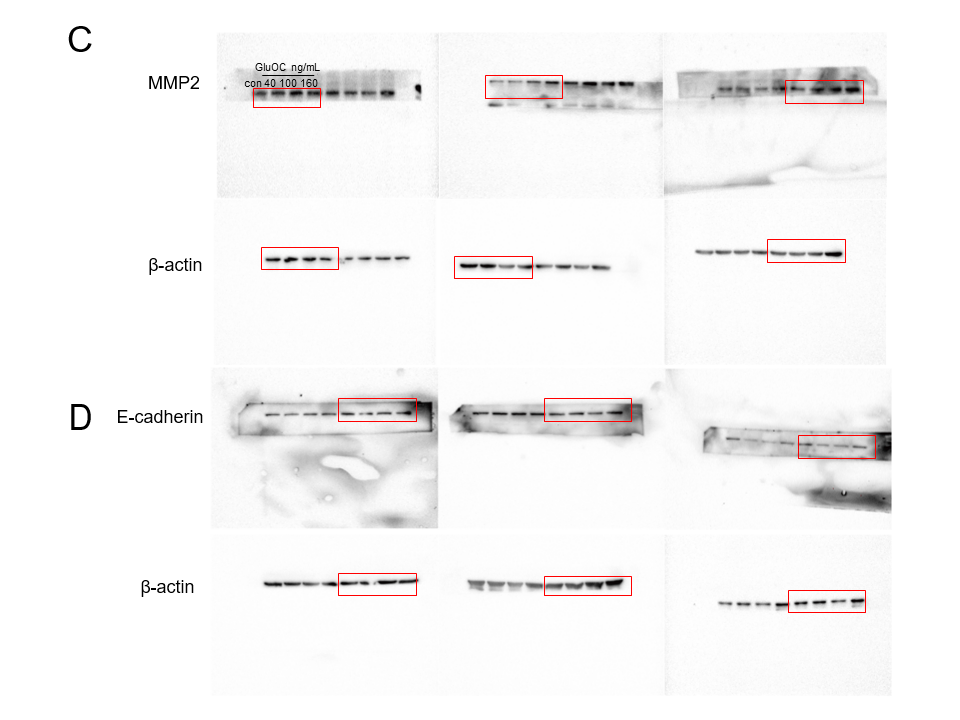


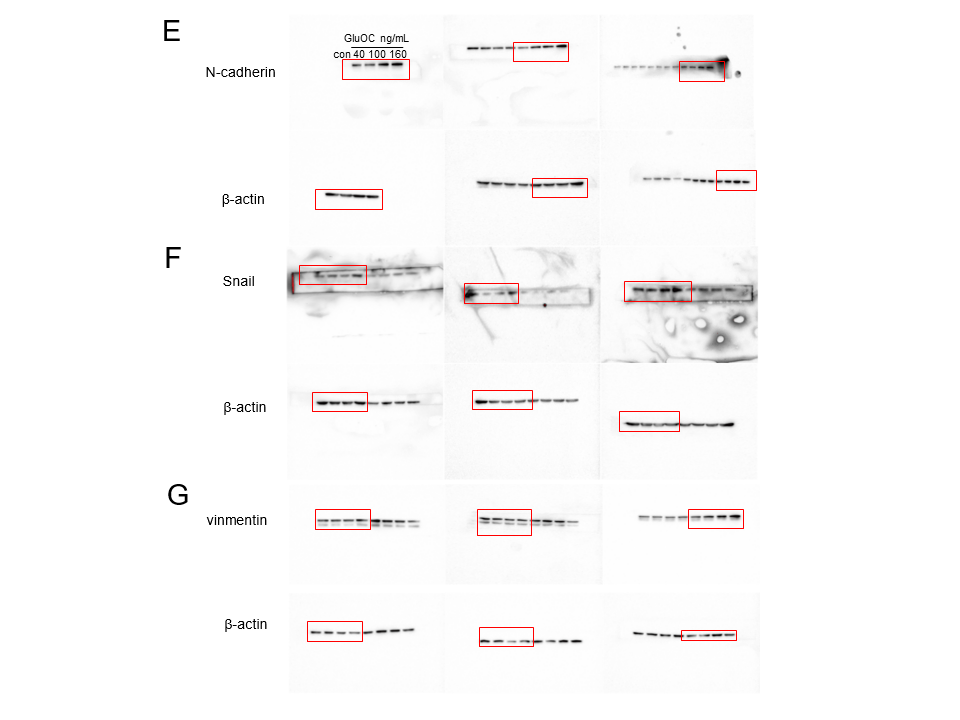


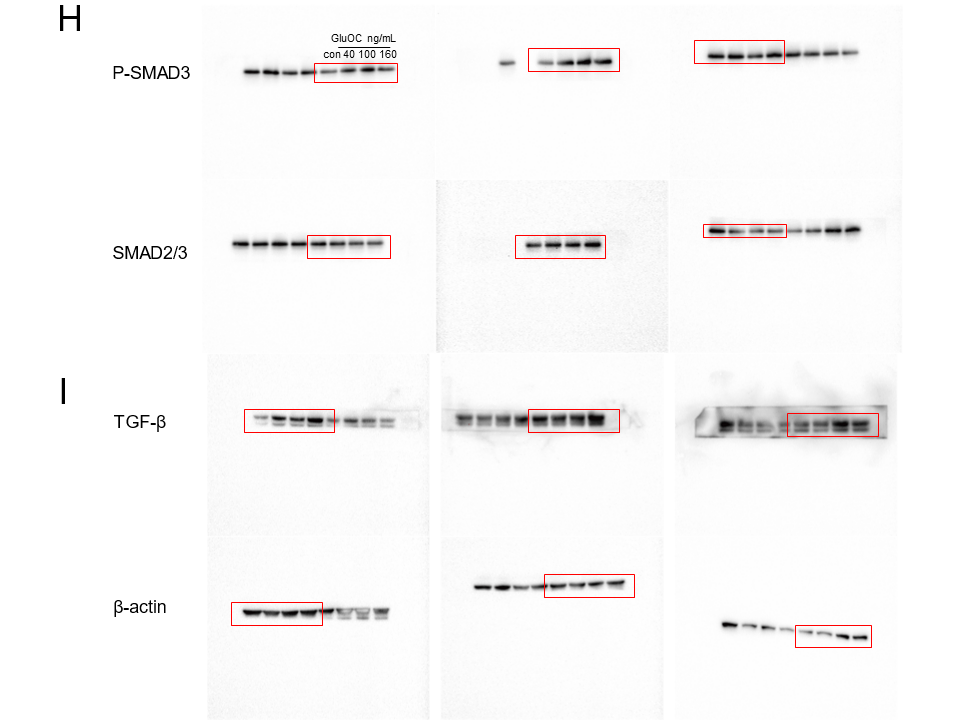


**Figure S1** Full-size images of Western blots of MDA-MB-231 cells. **A-B** Full-size image of Western blots to evaluate GluOC increases the proliferation of MDA-MB-231 cells shown in Figure 1b. **C** Full-size image of Western blots to evaluate GluOC increases MMP2 expression of MDA-MB-231 cells shown in Figure 3a. **D**, E, F, **G** are full-size images of Western blots for E-cadherin, N-cadherin, Snail, vimentin in MDA-MB-231 cells treated with GluOC at 40, 100, 160 ng/mL shown in Figure 4a, respectively. **H,** I, are full-size images of Western blots for P-SMAD3, SMAD2/3, TGF-β in MDA-MB-231 cells treated with GluOC at 40, 100, 160 ng/mL shown in Figure 6a, respectively. The red boxes indicate the cropped regions.


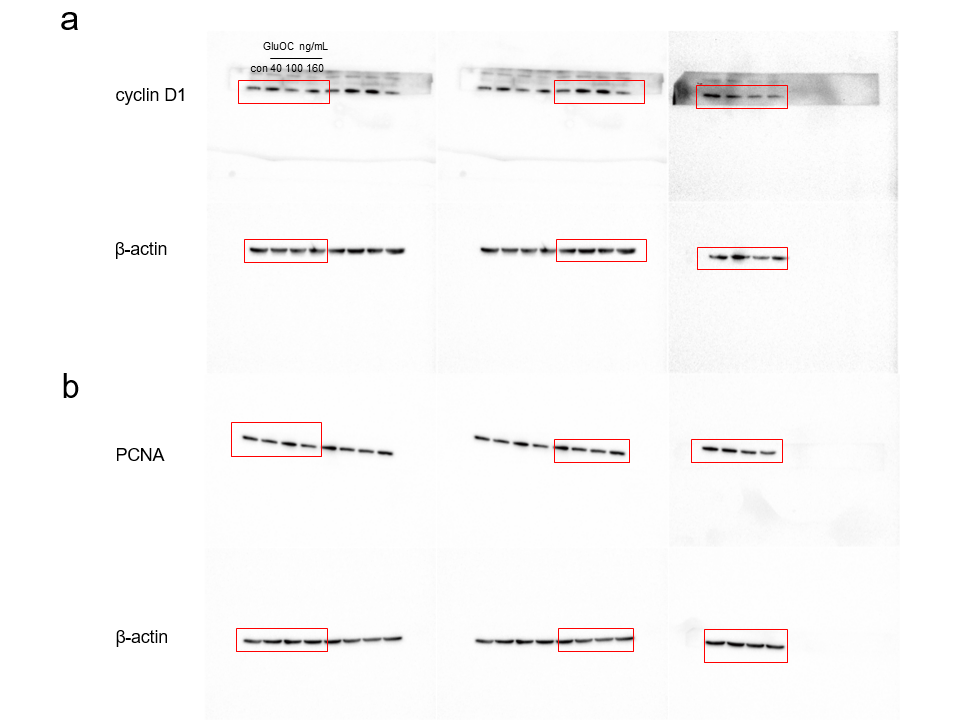


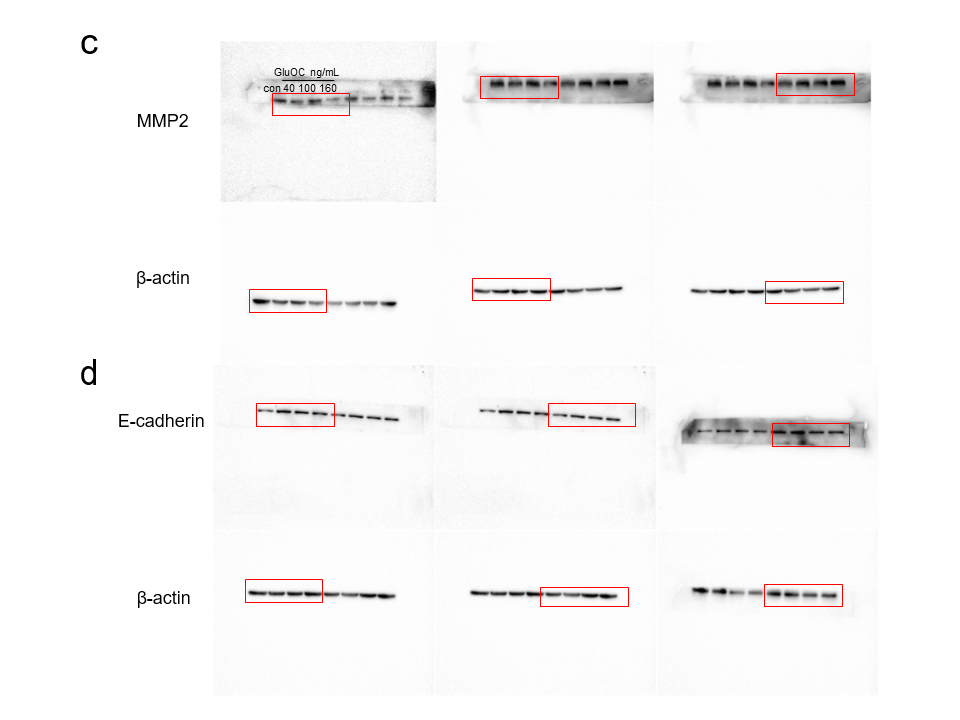


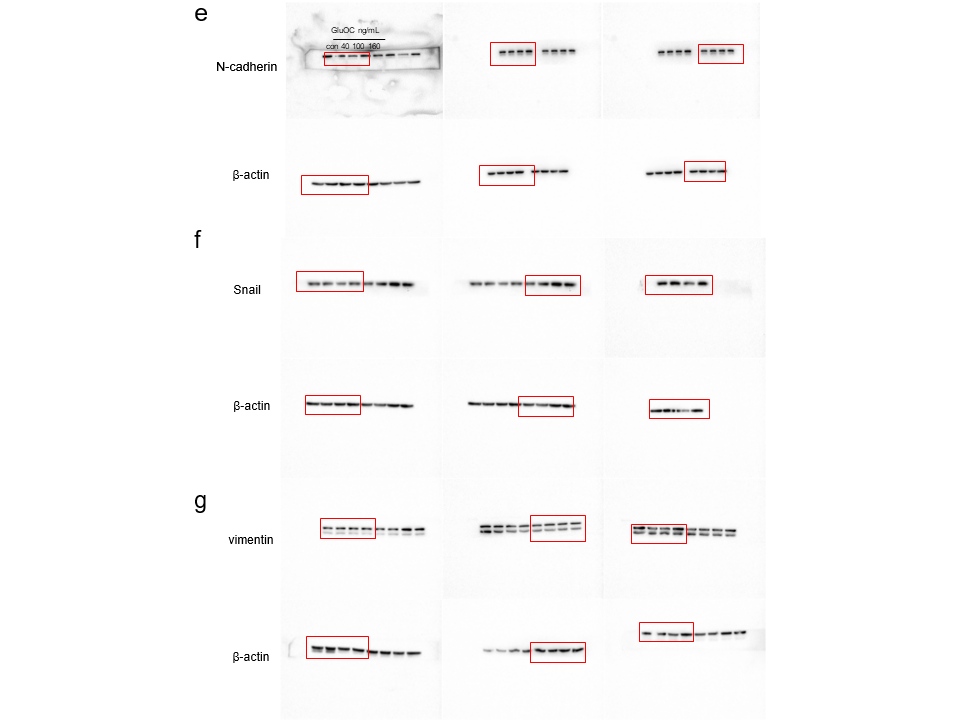


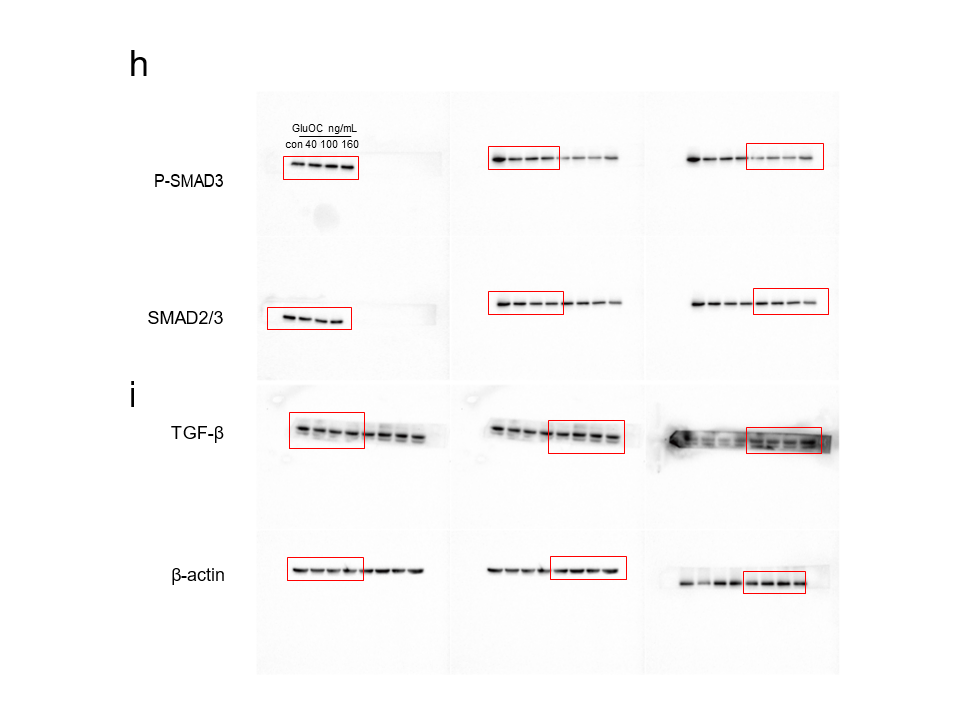


**Figure S2** Full-size images of Western blots of MCF7 cells. **a-b** Full-size image of Western blots to evaluate GluOC increases the proliferation of MCF7 cells shown in Figure 1c. **c** Full-size image of Western blots to evaluate GluOC increases MMP2 expression of MCF7 cells shown in Figure 3b. **d**, **e**, **f**, **g** are full-size images of Western blots for E-cadherin, N-cadherin, Snail, vimentin in MCF7 cells treated with GluOC at 40, 100, 160 ng/mL shown in Figure 4b, respectively. **h**, **i**, are full-size images of Western blots for P-SMAD3, SMAD2/3, TGF-β in MCF7 cells treated with GluOC at 40, 100, 160 ng/mL shown in Figure 6b, respectively. The red boxes indicate the cropped regions.


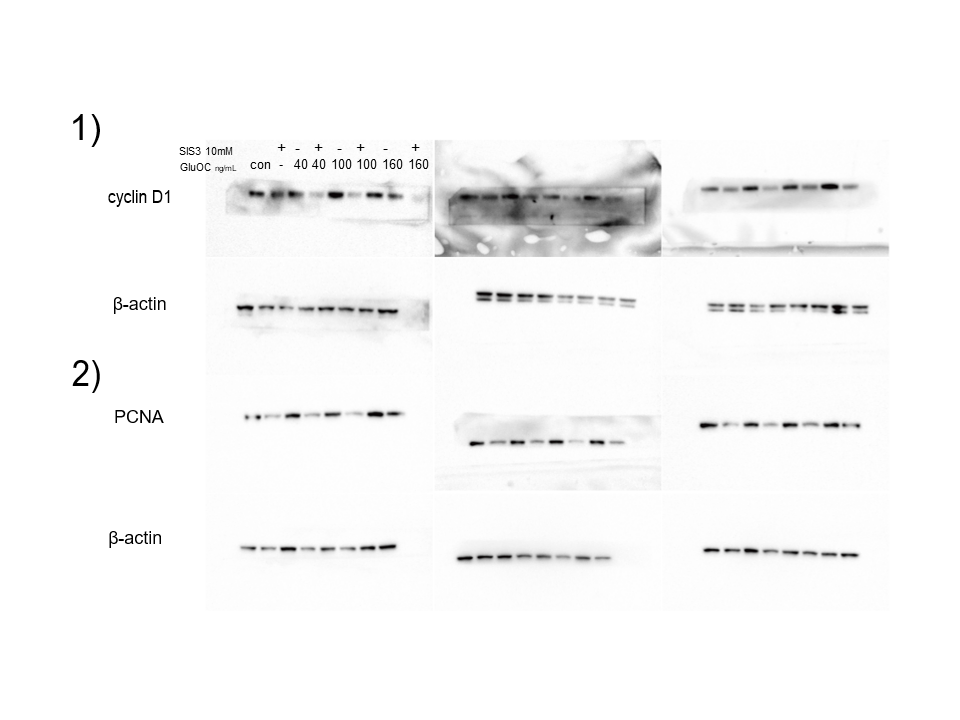


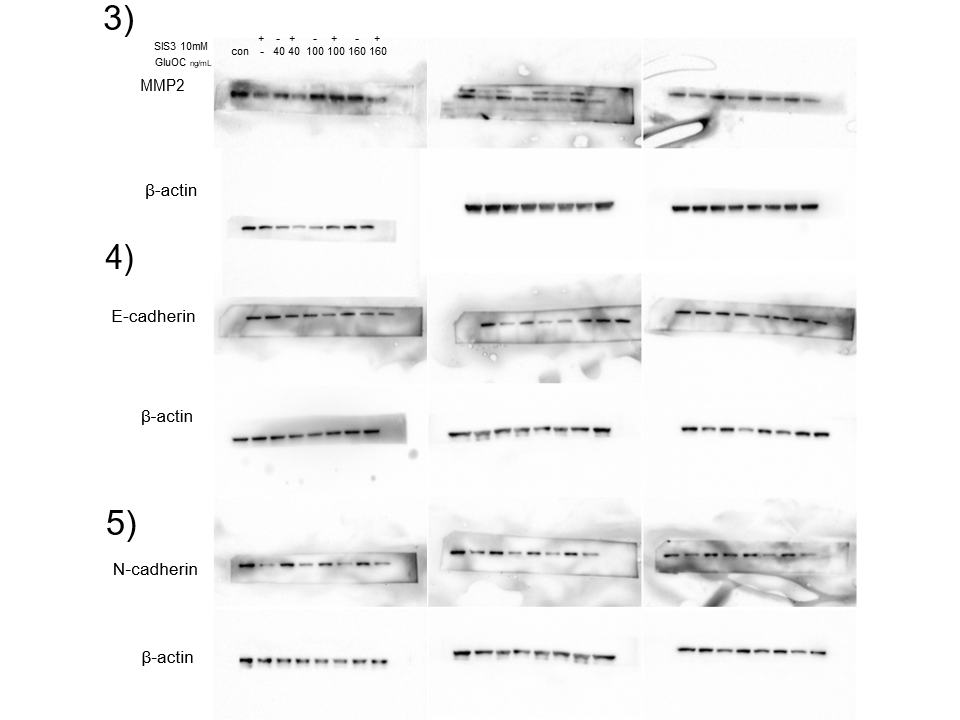


**Figure S3** Full-size images of Western blots of MDA-MB-231 cells. **1)-2)** After adding SIS3 and different concentrations of GluOC (40, 100, 160 ng/mL), full-size image of Western blots to evaluate GluOC increases the proliferation of MMDA-MB-231 cells by TGF-β/SMAD3 pathway shown in Figure 7a, respectively. 3) After adding SIS3 and different concentrations of GluOC (40, 100, 160 ng/mL), full-size image of Western blots to evaluate GluOC increases MMP2 expression of MMDA-MB-231 cells by TGF-β/SMAD3 pathway shown in Figure 8a. 4), 5) After adding SIS3 and different concentrations of GluOC (40, 100, 160 ng/mL), full-size image of Western blots to evaluate GluOC increases E-cadherin and N-cadherin expression of MMDA-MB-231 cells by TGF-β/SMAD3 pathway shown in Figure 9.
